# The Effect of Filler Content on the Tensile Behavior of Polypropylene/Cotton Fiber and poly(vinyl chloride)/Cotton Fiber Composites

**DOI:** 10.3390/ma13030753

**Published:** 2020-02-06

**Authors:** Elsadig Mahdi, Aamir Dean

**Affiliations:** 1 Department of Mechanical and Industrial Engineering, College of Engineering, Qatar University, Doha P.O. Box 2713, Qatar; elsadigms@qu.edu.qa; 2 Institute of Structural Analysis (ISD), Leibniz Universität Hannover, Appelstr. 9A, 30167 Hannover, Germany; 3 Elasticity and Strength of Materials Group, School of Engineering, Universidad de Sevilla, Camino de los Descubrimientos s/n, 41092 Seville, Spain

**Keywords:** natural composites, cotton fiber, material characterization, mechanical properties

## Abstract

This paper investigates the effect of filler content on the mechanical properties of cotton fiber (CF) on the CF/PP and CF/PVC composites under quasi-static loading. For this purpose, experimental tensile tests were carried out on dog-bone specimens, cut out from hot and cold press molded square plates of different fiber weight contents. The results obtained show that the filler content appears to have a strong influence on mechanical energy absorption, and failure characteristics. It was also found that the stiffness for both sets of material increases with the addition of filler. On the other hand, the ductility for both sets of the material increases with the addition of filler. The microscopic morphology study indicates that CF/PP possesses a glossy surface appearance compared to CF/PVC, which possesses a porous surface. Micro-scale damage characteristics from tensile tests indicate that material experienced shear failure, matrix cracking, fiber breakage, fiber fracture, and fiber pullout. The phenomenon of matrix crazing experienced by CF/PP composites was also observed.

## 1. Introduction

The use of fiber-reinforced polymers (FRP) in the aerospace and automotive industry is constantly increasing because of their inherent advantages compared to traditional materials. The increase in usage is not only reported in the aforementioned applications but also in many other technical applications, especially where high strength and stiffness are demanded but with low component weight [1,2]. However, classical FRP composites face challenging problems with their reuse and recycling, mainly due to the compounding of miscellaneous and typically very stable fibers and matrices [3,4]. An alternative option is to construct composites from materials made of renewable resources that involve economically and ecologically acceptable manufacturing technologies. For example, the use of natural fibers, which has recently attracted increased attention. In addition to their environmental benefits, the potential advantages of natural fibers are the plentiful supply of raw materials from renewable resources rather than from fossil sources and their low cost.

Natural fibers can be divided into mineral, animal, and plant fibers. Many authors have investigated different natural fibers for use in composites; see refs. [5,6,7,8,9,10,11,12,13,14]. As many of the plant fiber composites have similar properties to those of synthetic glass fiber composites, plant fibers have attracted the attention of researchers among the various natural fibers. Natural fibers are also light and provide excellent acoustic and thermal insulation. In ref. [15] it was shown that natural fiber-reinforced plastics are viable, light, inexpensive materials for motorcar door panels and are used in Mercedes Benz A-class and Ford model U hybrid-electric concept cars. Other possible applications include aerospace, biodegradable packaging, frames, and load-bearing applications [15,16]. Based on this, there is a growing interest in the properties of cellular materials for their use in impact energy-absorbing structures, and they have been made to work by refs. [17,18,19].

Plant fibers are composed of cellulose, hemicellulose, and lignin. Depending on their source they may generally be categorized as bast fibers (jute, flax, hemp, ramie, and kenaf), leaf fibers (abaca, sisal, and pineapple), seed fibers (coir, cotton, and kapok), core fibers (kenaf, hemp, and jute), grass and reed fibers (wheat, corn, and rice), and all other types (wood and roots) [20]. Cotton fibers are natural hollow fibers; they are soft, cool, breathable fibers, and are absorbent. Although cotton fibers have very promising physical properties, cotton fiber composites (with cotton fibers as fillers) have received limited attention from the scientific community and most of the existing investigations regarding cotton fibers have been devoted to cotton fabric composites [4].

Many researchers have investigated the mechanical properties of cotton fiber composites; see refs. [16,21,22,23,24,25,26,27,28,29,30]. However, most of the aforementioned studies were only focused on different aspects of material properties or using thermosets as the matrix. In this study a cheap and eco-friendly cotton fiber (CF) was incorporated with popular commodity plastics such as polypropylene (PP) and poly(vinyl chloride) (PVC). By using thermoplastics as a matrix, the inherent brittleness of thermosetting resins was reduced. Therefore to justify the points that have been presented, the focus was shifted to addressing the question, "Does filler content affect the mechanical properties of PVC/CF and PP/CF composites?" Accordingly, cotton fiber composite specimens or test coupons were fabricated with different filler contents. The cotton fiber composites were characterized by measuring their mechanical properties. The energy absorption capability of the material is measured. The effects of matrix types on the mechanical properties and energy absorption capabilities were studied. The microscopic morphology and damage characteristics were investigated.

## 2. Experimental Methodology

This section outlines the methodology being applied to carry out the experimental investigation.

### 2.1. Materials

The material used can be divided into two types, the matrix materials and the fiber materials. The former consist of two types, these are polypropylene (PP) and poly(vinyl chloride) (PVC). The latter is the cotton fiber (CF), which is used as a reinforcing material.

The polypropylene and poly(vinyl chloride) used were purchased from the Titan Group (M) Ltd. (Saskatoon, SK, Canada) PP has a density of 0.90 g/mL and a melt flow index specified according to ASTM D 1238 as 30 g/10 min, while the density of the PVC is 1.45 g/mL and its melt flow index is 15.7 g/10 min. The reported material properties of the matrices were provided by the manufacturer.

**Figure 1:**
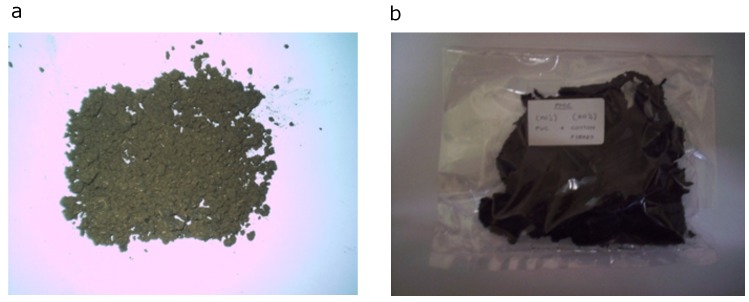
(**a**) Cotton fiber before being incorporated into polymers, and (**b**) a molded sample from the blending machine.

The cotton used here was brought from Sudan. It was dried, picked (seedless), and carded (i.e., pure cotton). It is worth mentioning that since the cotton used was dried, the moisture content was insignificant. A PM100CM model ball mill was used to cut the cotton into very fine sizes. First, the machine was run at 600 RPM for 10 min to get the fine cotton. Once the 10 min was over, the cotton was filtered using 3 sieves, one at 450 microns, one 80 microns, and one 25 microns. The excess cotton was returned into the machine to be ground again. The steps were repeated until enough cotton was collected. The size obtained was 425 microns. The cotton had a density of 1.5 g/cm^3^, an elongation of 7.9%, a tensile strength of 410 MPa, and Young’s modulus of 8.3 MPa. The physical appearance of cotton fibers is depicted in Figure 1a.

### 2.2. Experimental Design and Procedure

Herein, the sequences of samples preparation and the step by step procedure used to conduct the experiments are described in detail.

#### 2.2.1. Preparation of Specimens

The cotton fiber, PP, and PVC mixture were prepared according to the formulation given in Table 1.

#### 2.2.2. Molding of Specimens

**Figure 2:**
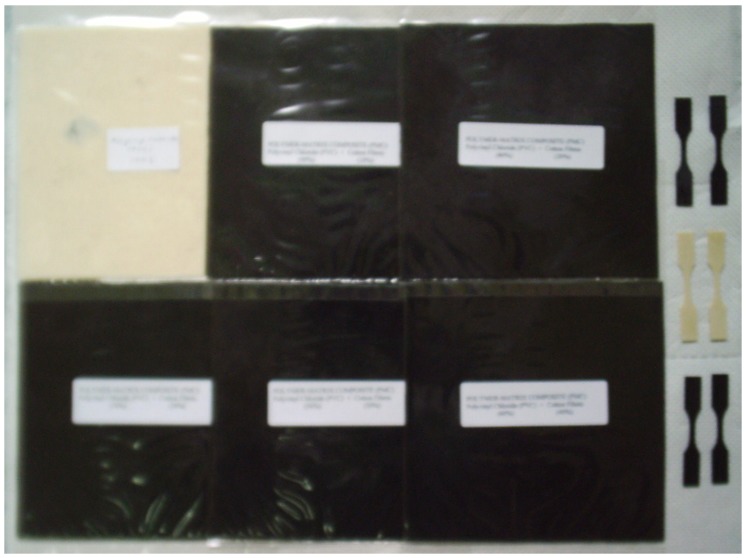
Set of pressed PVC specimens.

The cotton fiber composites specimens were prepared using a Thermo Haake PolyDrive extruder with Rheomix R600/610. The melt blending was carried out at 180 °C for both PP and PVC. The rotor speed was set to 100 rpm. The detailed steps were as follows. First, the weighted polymer was filled inside the mixing chamber with the use of the funnel tube. Then, the matrix was left to melt for 5 min. Next, the weighted cotton fiber was added into the mixing chamber together with the melted polymer. The mixing was stopped after the preset duration of 10 min. Finally, the specimens were removed from the mixing chamber while still at the set temperature. These steps were repeated for the remaining predetermined filler content as indicated in Table 1. The composite compounds obtained from melt blending were then compression-molded into 1 mm thick square plates under a pressure of 150 kg cm^−2^ at 190 °C for 2 min of pre-heating and 5 min of a full pressing cycle. The sheets were immediately cooled between two plates of cold-pressed at 25 °C for a 5 min cooling cycle. The end result can be seen in Figure 2.

It is worth noting that the purpose of this work was to make cost-effective, cotton fiber-reinforced thermoplastic composites. Therefore, neither intentionally using coupling agents nor a compatibilizer was permitted.

#### 2.2.3. Cutting of Specimens

**Figure 3:**
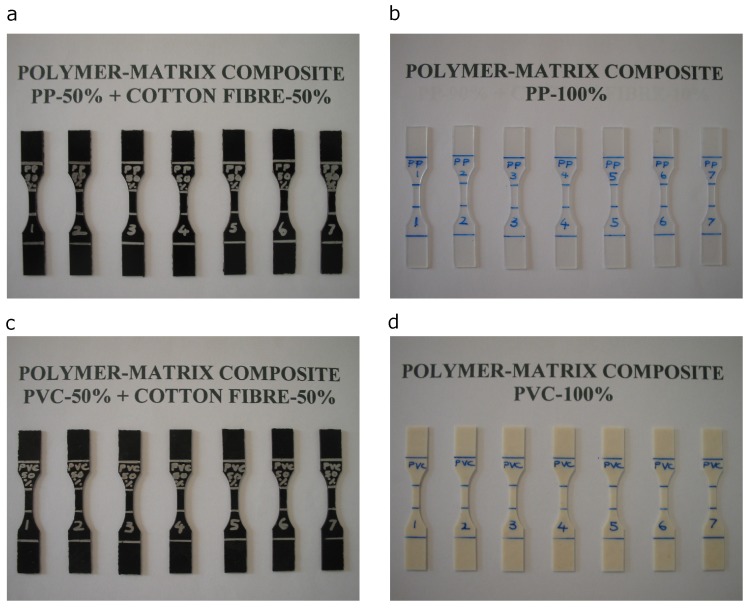
Selected set of dumbbell-shaped test coupons: (**a**) PP-50%/CF-50%, (**b**) PP-100%, (**c**) PVC-50%/CF-50%, and (**d**) PVC-100%.

Dumbbell-shaped test coupons were cut out from these sheets in accordance with ASTM D638—type 5; see Figure 3. The specimen’s gauge length was marked at 10 mm contrary to the specified gauge length of 7.62 mm. This was done to ease gauge length markings on specimens. The validity of data would not be affected by this change of gauge length, as it still lay within the length of a narrow section of the dumbbell-shaped test coupons. The test coupons were then measured for averaged gauge thickness using a Mitutoyo thickness gauge.

#### 2.2.4. Testing of Specimens

Tensile tests were carried out on INSTRON universal testing machine, using a load cell of 5 kN. The crosshead speed was set to 5 mm/min. The grip distance was set to 35 mm. The tensile tests were conducted inside a closed area, which maintained a temperature of 25 °C and humidity of 50%. In total, nine test specimens were used for the tensile test and the average of five results was taken as the resultant value. The detailed steps of running the tensile tests were as follows. At first, the width and thickness of each dumbbell test coupon were measured with a Mitutoyo thickness gauge to the nearest 0.025 mm at three points along the narrow sections and the average of the gauge thickness was calculated. The specimen was then placed in the grips of the universal testing machine. The grip distance was set to 35 mm and the aligning of the longitudinal axis of the specimen with the grips was done with the marked aligning distance on the specimens pre-calculated beforehand to improve accuracy. After the grip was tightened hydraulically, the built-in extensometer was calibrated to zero initial displacements and the average thickness was then input into the machine computer software. The test results were then automatically recorded. The same steps were then repeated for the remaining prepared test coupons. In order to study the morphological structures of CF/PP and CF/PVC composites, specimens that were taken from the fractured surfaces have been examined using an optical microscope.

### 2.3. Approach of Data Analysis

Herein, a compact explanation regarding the data analysis approach used is presented. As mentioned before, nine test specimens were used for the tensile test and the average of five results was taken as the resultant value. Thus the value of maximum stress, Young’s modulus, and elongation at break are the average values obtained from these specimens. A comparison chart was plotted to describe these properties. Consequently, the load and displacement data obtained were averaged by taking values of three specimens from each group of filler content to produce a load-displacement figure for each group. Energy absorption values were obtained from the calculation of previous load and displacement data using ANSYS engineering software. Finally, the morphologies of the CF composites were examined using an optical microscope.

According to Godwin [31], there are four criteria or factors to consider for a valid and reliable tensile tester; namely, its data acquisition, grips, alignment, and strain measurement. It is worth mentioning that the INSTRON testing machine used met these criteria.

## 3. Results and Discussion

Herein, the results obtained from experimental tests are presented and discussed in detail.

### 3.1. Mechanical Properties and Characteristics

This section presents the results obtained from the experimental data of the tensile tests. Herein, the measurements on some selected samples are analyzed. However, Figures 6–9 show the results of all tested cotton fiber reinforced PP/PVC composites.

#### 3.1.1. Pure Polypropylene (PP-100%)

**Figure 4:**
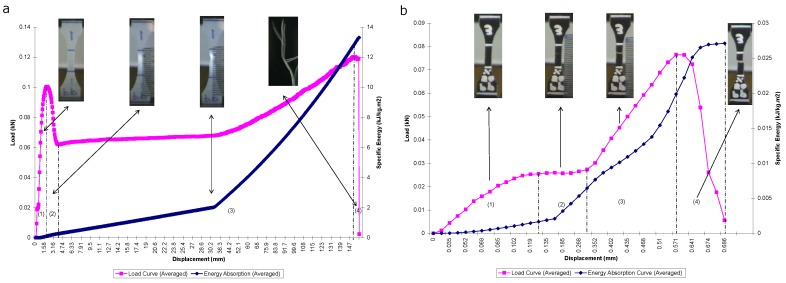
Load and energy absorption versus displacement: (**a**) PP-100% and (**b**) PP-50%/COTTON FIBER-50%.

From the tensile test conducted, the load-displacement curve obtained for PP100% is depicted in Figure 4a. It can be seen that in Region (1), the load increased linearly with displacement throughout this region. As the displacement increased, the load increased beyond the proportional limit until it reached a maximum point of elastic limit at about (1.909 mm, 0.1004 KN). A slight increase in load above the elastic limit resulted in a breakdown of the material and caused it to deform permanently. This is indicated by Region (2). It can be seen from the curve that the elastic-plastic transition occurred abruptly in what is the so-called yield point phenomenon. When yielding ended, a further load that was applied to the specimen resulted in a curve that rose until it reached a maximum load (or tensile strength) at about (150.8 mm, 0.1202 KN); see Region (3). At the point of maximum load, the cross-sectional area began to decrease in a localized region of the specimen. As a result, a constriction or "neck" occurred in this region, as displayed by the insets. The load-displacement diagram then continued its slight curve downward until the specimen broke at the fracture load.

It can be seen from Figure 4a that the energy absorption curve increases steadily, up to the point of specimen failure. Within Region (1) to Region (3), the maximum energy absorbed, i.e., initiation energy $E_{i}$, corresponds to the maximum load of the load-displacement figure at 12.96 kJ/kg m^2^. Beyond this region and until the endpoint of catastrophic failure, the energy absorbed, i.e., propagation energy $E_{p}$, is 0.3501 kJ/kg m^2^. The total energy absorption $E_{t}$ is 13.31 kJ/kg m^2^. The resilience energy is very much small in quantity compared to the total energy absorbed. This is due to the fact that plastic deformation is much higher than the elastic properties. Moreover, the total area under the load-displacement curve was found to be decreased as the filler content increased.

The value of maximum stress resulting from the tensile test was automatically obtained from the machine’s software, which corresponds to the value of the maximum load. The maximum stress was shown to be 35.841 MPa. The average value of Young’s modulus was calculated to be 388.042 MPa. The elongation at break obtained from the readings of gauge length elongation of the specimen is 850.00%.

#### 3.1.2. PP-50%/CF-50%

The load-displacement curve obtained for PP-50%/CF-50% is depicted in Figure 4b. The figure shows a similar linear property of the load-displacement curve within Region (1). The elastic limit was observed to be approximately at (0.1102 mm, 0.0247 KN). The specimen was permanently deformed as the load increased beyond that point. A steady flat line of load can be seen within Region (2) as the displacement increases. Within Region (3), the load gradually increased to the point of a maximum load as the specimen was further displaced. The point of maximum load is 0.0765 kN. Eventually, the load decreased to the point of fracture load, where the specimen was destructively failed. This is indicated in Region (4).

From Figure 4b, it can be seen that the energy absorption curve behaves similarly, as previously discussed, where the energy increased in a linear fashion within the Region (1) and kept increasing throughout the initiation phase. The $E_{i}$ value was taken to be 0.01988 kJ/kg m^2^, whereas within the propagation phase, the specimen continued to absorb energy up to the point of $E_{t}$ being equal to 0.027129 kJ/kg m^2^. $E_{p}$ is equal to 0.00725 kJ/kg m^2^.

From the recorded results, the average maximum tensile strength for PP-50%/CF-50% equals 18.749 MPa. The average modulus of elasticity value is 708.621 MPa, and the value of elongation at break reads 10.00%. In general, most of the CF/PP composite materials displayed plastic deformation; however, the increase in filler content resulted in more brittle behavior. It is also obvious to observe that the Young’s moduli improved with filler content. This can be strongly attributed to the addition of more fiber reinforcements into the matrix. Overall, the maximum tensile strength and strain at break values were observed to decrease as filler content increased for the CF/PP composites. The reduction of the maximum strength indicates that cotton fiber debonded from the PP matrix before the material experienced profound plastic deformation. The debonding between the cotton fiber and the PP matrix is also dominating the yielding stage and lowering the yield strength.

#### 3.1.3. Pure Poly(Vinyl Chloride) (PVC-100%)

**Figure 5:**
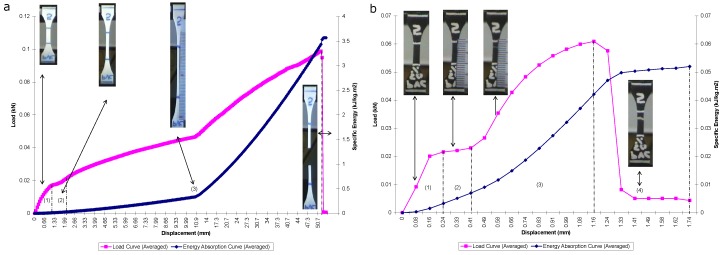
Load and energy absorption versus displacement: (**a**) PVC-100% and (**b**) PVC-50%/COTTON FIBER-50%.

The load-displacement curve obtained from the tensile test for PVC-100% is depicted in Figure 5a. Within Region (1) in the figure, it can be seen that the specimen experienced linear elastic behavior. As the displacement increased, the load increased beyond the proportional limit until it reached a maximum point of elastic limit at the end of Region (1). After this point, the material was permanently plastically deformed. This was indicated by Region (2). It was observed that the yielding phase for PVC was much shorter than that of PP. The specimen then continued to elongate with a gradual increase in load. Within Region (3), the further load applied to the specimen rose continuously to the point of the maximum load of (51.49 mm, 0.0988 KN). At this maximum load, a small constriction or necking began to form, as indicated by the inset. The load-displacement diagram then immediately curved downward until the specimen broke at the fracture load, as identified in Region (4). It was also observed that the phase within Region (3) was much longer for PVC than PP. This is maybe due to the inherently higher ductility of PVC material compared to PP.

It is observable from Figure 5a that the specific energy increased slowly with increased displacement. $E_{i}$ is taken to be 3.434 kJ/kg m^2^. As soon as the specimen enters the propagation phase, the energy increased to the point of fracture. The total absorbed energy $E_{t}$ equals 3.568 kJ/k m^2^ while $E_{p}$ is 0.1332 kJ/kg m^2^.

From the results obtained, the average maximum stress calculated for PVC-100% is 24.774 MPa, while the average modulus of elasticity is 40.104 MPa. The value of the average elongation at break from the recorded gauge length elongation is 206.67%.

#### 3.1.4. PVC-50%/CF-50%

The load-displacement relationship of Figure 5b shows that the tensile load increased linearly with displacement and reached the elastic limit at the value of approximately (0.2427 mm, 0.0216 KN). This is depicted by Region (1). After this stage, the specimen yielded plastically throughout Region (2). The load then increased until it reached the maximum point in the load-displacement diagram. The maximum load occurred at (1.159 mm, 0.0609 KN). This is depicted in Region (3). The specimen failed at the fracture load, which is indicated by Region (4).

Figure 5b shows that the energy absorption increased linearly throughout the initiation phase. The maximum point of energy absorbed within this region $E_{i}$ was 0.04212 kJ/kg m^2^, whereas $E_{t}$ was observed to be 0.0520 kJ/kg m^2^. Hence, $E_{p}$ was obtained as 0.009873 kJ/kg m^2^.

The average maximum stress was shown to be 13.372 MPa. After all nine samples had been tested, the average modulus of elasticity was obtained as 276.344 MPa. The recorded test results yielded a value of average elongation at break of 10.50%.

### 3.2. Data Analysis and Discussions

This section focuses on the interpretation and analysis of results obtained, and the discussions of interpreted results.

#### 3.2.1. Comparison of the Load–Displacement Relationship

**Figure 6:**
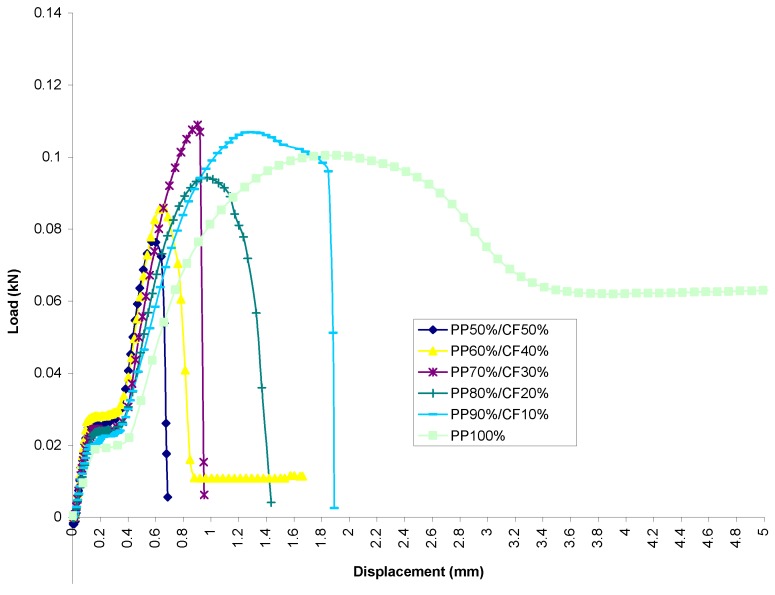
Load versus displacement for averaged curve of cotton fiber reinforced PP composites.

**Figure 7:**
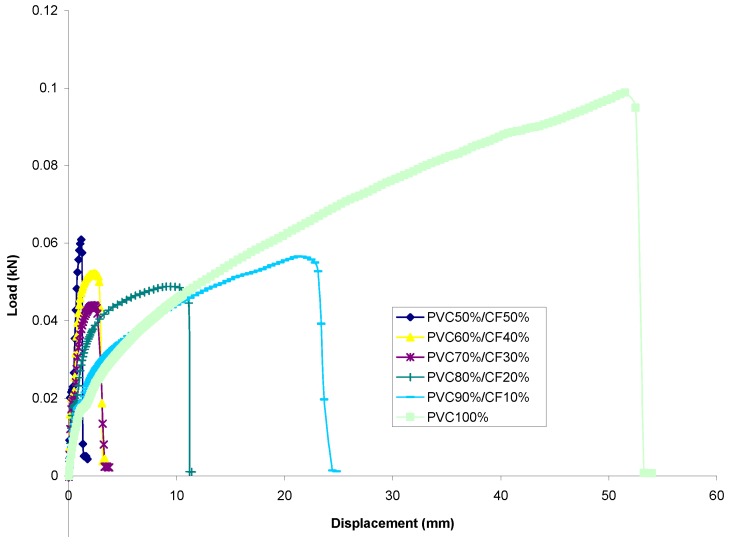
Load versus displacement for averaged curve of cotton fiber reinforced PVC composites.

- PP Set
  Figure 6 shows a combination of all averaged cotton fiber reinforced PP with different filler contents. It can be seen that all the percentages of filler addition to PP studied displayed atypical behavior for a polymer or even for most metals in terms of mechanical behavior when subjected to axial tensile load. Generally, the curve shows that as the percentage of filler content increases, the failure occurs at a shorter displacement interval; see refs. [32,33].
  It was observed that all material within the PP set showed a linear elastic behavior initially. From Figure 6, one can observe that the slopes within the proportional limits do not differ much from each other; i.e., moduli of elasticity do not differ greatly from each other. A similar observation was made in refs. [4,20].
  The figure shows that by filler addition, the specimens exhibit a smooth transition from elastic to plastic region compared to pure PP, which exhibits yield point phenomenon with upper and lower yield point. It also displays that the load transition from strain hardening to maximum load is different for each percentage of filler content. This is indicated by the different gradients of slopes, with specimens of PP-50%/CF-50% having the steepest slope and pure PP the least steep; see ref. [4].
  Furthermore, it was observed that the trend of the load-displacement curve from maximum to fracture load differed depending on filler content. The entire group of specimens experienced a smooth, bell-shaped-like transition to their fracture points with the exception of PP-80%/CF-20% and PP-100%, which failed abruptly after reaching the maximum load.
- PVC Set
  Figure 7 shows the load-displacement relationship of all the samples tested combined together. Similar to PP set, all samples within the PVC set show a typical behavior of mechanical characteristic curve for polymer when subjected to tensile load. This can be interpreted as showing that it is the matrix phase which contributes to this characteristic. Similarly, it was observed that the failure of the specimens occurs over a shorter duration with increased cotton fiber addition.
  Identical to the PP set, the figure displays that a representative percentage of specimens behaves in a linear elastic manner within the proportional limit. However, it was found that for each representative percentage of specimens, the modulus of elasticity appears to differ more compared to the PP set; see ref. [34].
  Contrary to samples of PP set, the load-displacement curve transition from maximum load to fracture load does not exhibit a bell-shaped-like transition. In fact, the specimens experienced a catastrophic drop immediately after reaching the point of maximum load.
  A significant point to mention here is that samples of PVC set show a shorter duration or a shorter interval of yielding phase compared to curves from PP set. The correlation of this phenomenon may be related to the brittleness or ductility property of the matrix.

#### 3.2.2. Comparison of the Energy–Displacement Relationship

**Figure 8:**
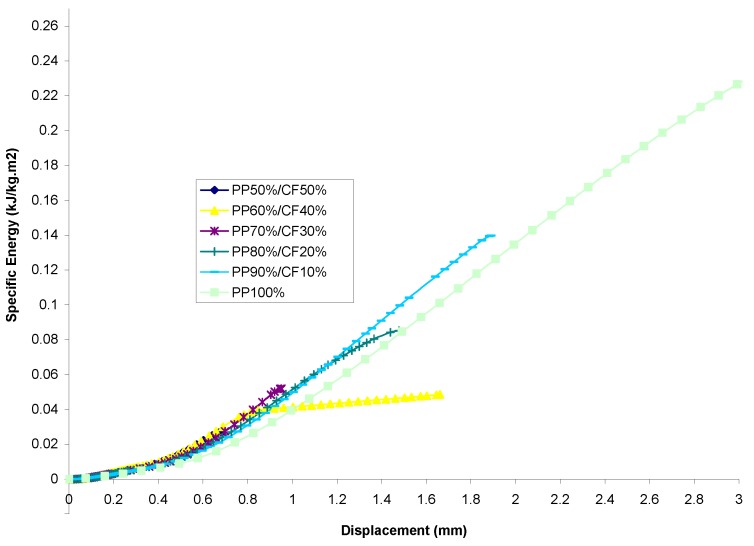
Energy versus displacement for averaged curve of cotton fiber reinforced PP composites.

**Figure 9:**
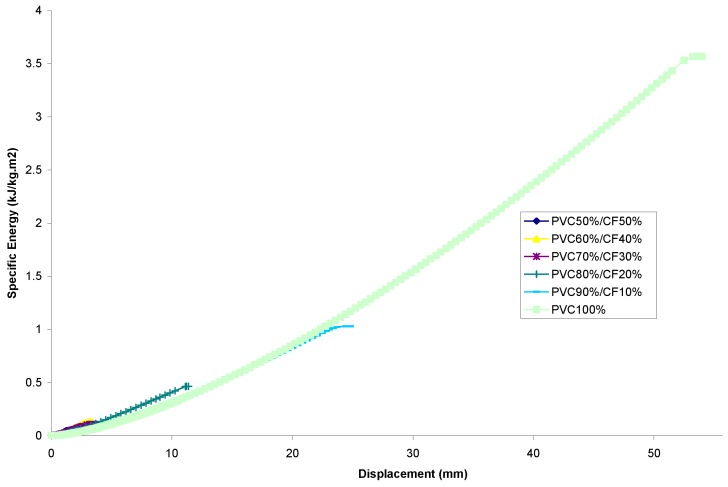
Energy versus displacement for averaged curve of cotton fiber reinforced PVC composites.

- PP Set
  Figure 8 shows the energy-displacement relationship of samples for the PP set. It can be seen that the specific energy of every sample increases linearly within the region of the initiation phase. All of the specimens exhibit a smooth, gradual increase in energy absorption through the propagation phase, up to the point of destructive failure of specimens, similar to the observation made in ref. [4].
  The figure also shows that the highest energy absorption value was found from PP-100%, whereas the lowest value of energy absorption was found to be possessed by PP-50%/CF-50%. Disregarding PP-100% from consideration and treating it as a controlled specimen, it was found that by filler addition, PP-90%/CF-10% is the optimum composite mixture to possess maximum energy absorption capability. The difference in percentage between the two extremities was calculated and found to be 415.1% higher than the lowest value.
- PVC Set
  Energy-displacement curves obtained from the experiment for the PVC set are depicted in Figure 9. It can be seen that the specific energy of all specimens increases in a linear manner within the initiation phase, similar to the PP set. However, the difference between these two sets lies in the transition from the initiation phase to the end of the propagation phase. The linearity of all the PP composites’ curves is maintained and conserved to the point of fracture. This characteristic from the author’s point of view is significant for the employment of design criteria that stressed a material having a predictable energy absorption capability.
  By considering the pure PVC (i.e., PVC-100%) as control samples, it was also observed that the highest energy absorption capability was possessed by PVC-90%/CF-10% as opposed to PVC-50%/CF-50%, which had the lowest value of energy absorption capability; see ref. [34]. The difference between the two in terms of percentage is 1882.7%.

#### 3.2.3. Comparison of Energy Profile Characteristics

Table 2 and Table 3 show that all representative filler content within PP set and PVC set exhibit larger initiation energy, $E_{i}$ than propagation energy, $E_{p}$. This complies with the findings in ref. [35] and shows that more energy was absorbed in order to initiate fracture in the form of micro buckling of fibers or in the form of debonding at the fiber-matrix interface. It was also observed that energy absorption capability decreases as filler (CF) content increases with the exception of one point in the PVC set profile where 40% of filler addition increases slightly over filler addition of 30%.

It can be clearly seen that the PVC set exhibits a larger energy absorption capability (total energy absorption) compared to the PP set. This shows that matrix type plays an important role in the energy absorption capability of composite materials; see ref. [12]. However, according to ref. [36] the total value of energy absorption does not provide much information about the fractured nature of a material. Therefore, to get a better understanding of the studied material, the so-called Ductility Index ($DI=\frac{E_{p}}{E_{i}}$) was evaluated.

The DIs of the PP set and PVC set are provided in Table 2 and Table 3. It can be seen that both the PP set and PVC set possess inherently low indices of ductility. This implies that both sets of materials are relatively brittle, and thus indicates that most of the energy was absorbed elastically; see ref. [36].

The corresponding Table 2 indicates that there are inconsistencies with the DI profile for the PP set. The profile increases initially and fluctuates for 20% and 30% of CF content; then, increases again for 40% CF content; and finally, fluctuates for 50% of CF addition. In contrast, the DI for PP profile exhibits an increasing trend of DI with increased CF content and it finally decreases slightly for CF content of 50%. However, the authors should refrain from jumping to a conclusion by inferring that the ductility increases with increased filler content. A comparison with ductility (elongation at break) data from the tensile test was done and is discussed in the next section.

#### 3.2.4. Tensile Strength and Filler Content Relationship

Table 4 shows that the average tensile strength of PP/CF composites decreases from 0% to 20% filler addition, increases slightly for 30% filler addition, and continues to decrease for the remaining amounts filler addition. As for PVC/CF composites, the results show a decreasing tensile strength to the point of 30% of filler addition before it increases slightly for the remaining percentage of filler addition. This complies with the observations made in ref. [37] that the incorporation of filler to a polymer matrix may increase or decrease the tensile strength of the resulting composite.

#### 3.2.5. Stiffness (Modulus of Elasticity) and Filler Content Relationship

Modulus of elasticity is one of the basic properties of composites, as the primary intention of filler incorporation is usually to increase the stiffness of the resultant material. Table 5 shows the modulus of elasticity of PP/CF composites and PVC/CF composites. As expected, the modulus of elasticity increases steadily with increasing filler content.

#### 3.2.6. Ductility (Elongation at Break) and Filler Content Relationship

Incorporation of CF filler into PP and PVC decreases the elongation at the break of the respective matrices. Table 6 shows that the elongation at break decreases with increasing filler content. This is a common observation with almost all filled polymer systems. The reduction in elongation took place due to the decreased deformability of rigid interphase between the CF and the matrix material; see ref. [37].

### 3.3. Microscopic Morphology and Damage Characteristics

Microscopic morphology examination of the CF composites was done for all of the samples within the PP and PVC set on specimens that were taken from the fractured surfaces. From the examination of PP/CF composite specimens under an optical microscope, it was observed that the morphological characteristics of each group of filler content were more or less the same. Thus, only representative specimens were included for discussion.

#### 3.3.1. Surface Morphology

**Figure 10:**
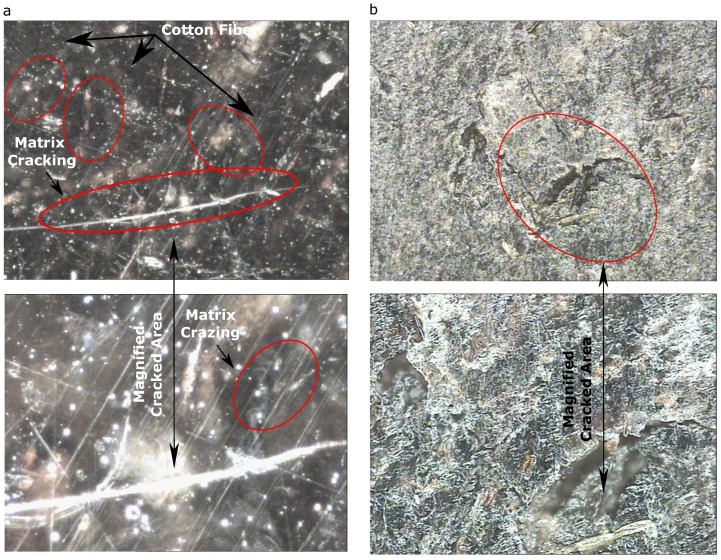
Representative samples (30% of filler addition) that show the surface of the material with 20× (top) and 50× magnification (bottom) taken after the tensile test and from the fracture surface for (**a**) PP/CF composites, and (**b**) PVC/CF composites.

- PP/CF composites
  Figure 10a shows a representation of PP/CF composites with regard to its surface characteristics. It can be observed that the CF fibers are discontinuous and randomly oriented. Therefore, in order to obtain comprehensive material properties at a microstructural level, the material should be analyzed further, considering anisotropy and heterogeneity.
  The figure shows that the material has a naturally occurring glossy finish upon molding. In certain design criteria that require a mirror-like finish, this characteristic is desirable as there will be no need employing a resin-rich outer surface.
  It can be seen that the so-called matrix crazing had occurred on the material surface, similar to the observations made by ref. [38]. It is also visible that fiber breakage had occurred along the matrix crack and within the matrix, as indicated in the figure.
- PVC/CF composites
  Figure 10b demonstrates the surface characteristics of PVC/CF composites. Similar to the PP set, it can be seen that the fibers are discontinuous and randomly oriented.
  Figure 10b shows that the surface of the specimen is quite porous. This differs from that of PP/CF composites, which showed a more of a glossy surface. It also can be seen that microcrack had occurred with a fiber breakage along the crack. The crack occurred probably because of the dry shrinkage of the sample during the experimental process.

#### 3.3.2. Damage Characteristics

**Figure 11:**
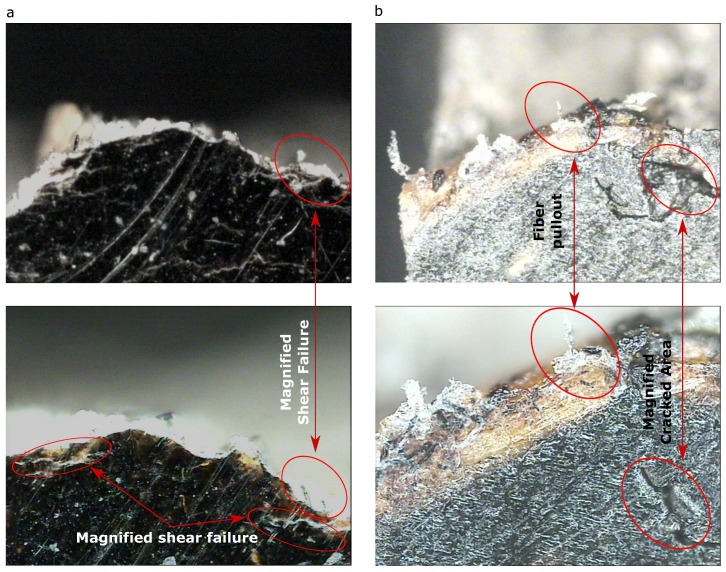
Representative samples (30% of filler addition) that show the failed material with 20× (top) and 50× magnification (bottom) taken after the tensile test and from the fracture surface for (**a**) PP/CF composites, and (**b**) PVC/CF composites.

- PP/CF composites
  From Figure 11a, composite failure along the width of the gauge shows that shear failure occurred. It can be seen clearly, in both figures, that fiber breakage and fiber fracture occurred along the breakage section. It was also observed that matrix cracking was prominent along the breakage section, as pointed out in the figure.
- PVC/CF composites
  Similar to the PP set, Figure 11b reveals that shear failure occurred. It can be observed that fiber breakage and fiber fracture, and fiber pullout, occurred along the breakage section. It was also observed that complex microcracks were prominent along the breakage section indicated.

## 4. Concluding Remarks And Outlook

Polypropylene/cotton fiber and poly(vinyl chloride)/cotton fiber composites samples were fabricated and tensile tested throughout the work. The effects of filler content on the mechanical properties (energy absorption, maximum stress, modulus of elasticity, and elongation at break) of polypropylene/cotton fiber and poly(vinyl chloride)/cotton fiber composites have been studied in this work. The following conclusion can be made regarding this work:

- Energy absorption capability decreases with increased filler (CF) content.
- Both PP/CF composites and PCV/CF composites possess inherently low ductility index, implying both composites are relatively brittle.
- The tensile strength (maximum stress) of both materials decreases with filler content, with the peculiar exceptions of 30% filler content for PP/CF composites and 40% and 50% filler content for PVC/CF content.
- The stiffness (modulus of elasticity) for both sets of material increases with the addition of filler.
- Ductility (elongation at break) decreases with increased filler content for PP/CF and PVC/CF composites. Hence, agreeing with DI calculated which generalized that both materials were relatively brittle.
- Microscopic morphology indicates that PP/CF possesses a glossy surface appearance compared to PVC/CF, which possesses a porous surface.
- Micro-scale damage characteristics from tensile tests indicate that material experienced shear failure, matrix cracking, fiber breakage, fiber fracture, and fiber pullout. It was also observed the phenomenon of matrix crazing experienced by PP/CF composites.

In order to gain a full view of the mechanical properties of the studied materials, it is suggested that the results obtained should be supplemented with results from other tests; e.g., impact strength, hardness, toughness, and DMTA tests.

It is also suggested that similar work should be done by adding compatibilizer into the CF composites to achieve good intermolecular bonding between the matrixes and the fiber phases. That is the case when natural hydrophilic fibers are added to hydrophobic matrices. The composites should be prepared following the recommendations drawn in ref. [39] and then analyzed further by regarding them as anisotropic and heterogeneous materials to obtain comprehensive material properties at a microstructural level.

In a next step, the sophisticated models developed in refs. [40,41] will be employed to numerically investigate the failure behavior of the studied materials.

## Figures and Tables

**Table 1:** Formulation for PP and PVC mixture with cotton fiber.

Fiber Content (wt%)	PP/PVC Content (wt%)	Net Weight of Fiber (g)	Net Weight of PP/VC (g)
0	100	0.0	44.0
10	90	4.4	39.6
20	80	8.8	35.2
30	70	13.2	30.8
40	60	17.6	26.4
50	50	22.0	22.0

**Table 2:** Initiation energy $E_{i}$, propagation energy $E_{p}$, and ductility index DI for the PP set with different filler content, CF.

CF (wt%)	$E_{i}$ (kJ/kg m^2^)	$E_{p}$ (kJ/kg m^2^)	DI
0	12.955±1.414	0.350±0.016	0.027
10	0.079±0.060	0.139±0.002	0.765
20	0.048±0.006	0.0364±0.008	0.742
30	0.0484±0.009	0.003±0.019	0.069
40	0.027±0.002	0.021±0.001	0.766
50	0.0198±0.0008	0.007±0.001	0.364

**Table 3:** Initiation energy $E_{i}$, propagation energy $E_{p}$, and ductility index DI for PV set with different filler content, CF.

CF (wt%)	$E_{i}$ (kJ/kg m^2^)	$E_{p}$ (kJ/kg m^2^)	DI
0	3.434±0.134	0.133±0.013	0.038
10	0.896±0.070	0.134±0.001	0.150
20	0.363±0.003	0.102±0.002	0.282
30	0.079±0.001	0.027±0.001	0.350
40	0.092±0.001	0.036±0.0185	0.398
50	0.042122±0.0001288	0.009873±0.0000972	0.234398

**Table 4:** Tensile strength for PP and PVC set with different filler content, CF.

CF (wt%)	Tensile Strength PP (MPa)	Tensile Strength PVC (MPa)
0	35.80±2.10	24.80±1.14
10	28.0±1.50	14.10±2.88
20	24.60±1.12	11.80±0.35
30	27.00±0.12	11.80±0.045
40	23.80±0.15	12.50±0.023
50	18.80±0.20	13.40±0.13

**Table 5:** Modulus of elasticity for PP and PVC set with different filler content, CF.

CF (wt%)	Modulus of Elasticity PP (MPa)	Modulus of Elasticity PVC (MPa)
0	388.0±2.50	39.0±3.00
10	440.0±3.30	78.0±3.60
20	640.0±4.40	126.0±1.90
30	641.0±6.80	188.0±0.80
40	700.0±7.70	244.0±2.0
50	709.0±3.10	280.0±1.90

**Table 6:** Average elongation at break for PP and PVC set with different filler content, CF.

CF (wt%)	Elongation PP (%)	Elongation PVC (%)
0	850.00±1.50	206.70±2.00
10	15.70±2.20	105.00±0.60
20	15.70±1.20	57.00±1.90
30	10.00±2.10	35.00±0.30
40	10.00±1.20	13.00±0.2
50	10.00±0.10	10.50±2.10
